# Genetic homogeneity of goat malaria parasites in Asia and Africa suggests their expansion with domestic goat host

**DOI:** 10.1038/s41598-018-24048-0

**Published:** 2018-04-11

**Authors:** Morakot Kaewthamasorn, Mika Takeda, Tawee Saiwichai, Jesse N. Gitaka, Sonthaya Tiawsirisup, Yuhei Imasato, Ehab Mossaad, Ali Sarani, Winai Kaewlamun, Manun Channumsin, Suchart Chaiworakul, Wichit Katepongpun, Surapong Teeveerapunya, Jarus Panthong, Dominic K. Mureithi, Saw Bawm, Lat Lat Htun, Mar Mar Win, Ahmed Ali Ismail, Abdalla Mohamed Ibrahim, Keisuke Suganuma, Hassan Hakimi, Ryo Nakao, Ken Katakura, Masahito Asada, Osamu Kaneko

**Affiliations:** 1 Veterinary Parasitology Research Group, The Veterinary Parasitology Unit, Department of Pathology, Faculty of Veterinary Science, Chulalongkorn University, Bangkok, 10330 Thailand; 2 Animal Vector-Borne Disease Research Group, The Veterinary Parasitology Unit, Department of Veterinary Pathology, Faculty of Veterinary Science, Chulalongkorn University, Bangkok, 10330 Thailand; 3 Department of Protozoology, Institute of Tropical Medicine (NEKKEN), Nagasaki University, 1-12-4 Sakamoto, Nagasaki, 852-8523 Japan; 4 Department of Parasitology and Entomology, Faculty of Public Health, Mahidol University, Bangkok, 10400 Thailand; 5 Department of Clinical Medicine, Mount Kenya University, PO Box, 342-01000 Thika, Kenya; 6 Laboratory of Parasitology, Graduate School of Infectious Diseases, Faculty of Veterinary Medicine, Hokkaido University, Sapporo, 060-0818 Japan; 7 Department of Pathology, Parasitology and Microbiology, College of Veterinary Medicine, Sudan University of Science and Technology, P.O. Box 204, Khartoum, Sudan; 8 Department of Clinical Science, University of Zabol, Veterinary Faculty, PO box +9861335856 Zabol, Iran; 9 School of Agricultural Resources, Chulalongkorn University, Phayathai Rd., Pathumwan, Bangkok, 10330 Thailand; 10 Faculty of Veterinary Medicine, Rajamangala University of Technology Tawan-Ok 43 Moo 6 Bangpra, Sriracha District, Chonburi, 20110 Thailand; 11 Faculty of Agriculture and Natural Resources, Rajamangala University of Technology Tawan-Ok 43 Moo 6 Bangpra, Sriracha District, Chonburi, 20110 Thailand; 12 Livestock Office of Phetchaburi Province, Department of Livestock Development, Phetchaburi, 76000 Thailand; 13 Livestock Office of Kaeng Krachan District, Department of Livestock Development, Phetchaburi, 76180 Thailand; 14 Department of Animal Health and Production, School of Pure and Applied Sciences, Mount Kenya University, P O Box, 342-01000 Thika, Kenya; 15 Department of Pharmacology and Parasitology, University of Veterinary Science, Nay Pyi Taw, 15013 Myanmar; 16 Rector Office, University of Veterinary Science, Nay Pyi Taw, 15013 Myanmar; 17 Abrar Research and Training Centre, Abrar University, Mogadishu, Somalia; 18 National Research Center for Protozoan Diseases, Obihiro University of Agriculture and Veterinary Medicine, Obihiro, Hokkaido 080-8555 Japan; 19 Research Center for Global Agromedicine, Obihiro University of Agriculture and Veterinary Medicine, Obihiro, Hokkaido 080-8555 Japan; 20 Graduate School of Biomedical Sciences, Nagasaki University, 1-12-4 Sakamoto, Nagasaki, 852-8523 Japan

## Abstract

*Plasmodium* was first identified in a goat in Angola in 1923, and only recently characterized by DNA isolation from a goat blood sample in Zambia. Goats were first domesticated in the Fertile Crescent approximately 10,000 years ago, and are now globally distributed. It is not known if the *Plasmodium* identified in African goats originated from parasites circulating in the local ungulates, or if it co-evolved in the goat before its domestication. To address this question, we performed PCR-based surveillance using a total of 1,299 goat blood samples collected from Sudan and Kenya in Africa, Iran in west Asia, and Myanmar and Thailand in southeast Asia. *Plasmodium* DNA was detected from all locations, suggesting that the parasite is not limited to Africa, but widely distributed. Whole mitochondrial DNA sequences revealed that there was only one nucleotide substitution between Zambian/Kenyan samples and others, supporting the existence of a goat-specific *Plasmodium* species, presumably *Plasmodium caprae*, rather than infection of goats by local ungulate malaria parasites. We also present the first photographic images of *P*. *caprae*, from one Kenyan goat sample.

## Introduction

Malaria is a mosquito-borne disease caused by intracellular protozoan parasites of the genus *Plasmodium*. In addition to human malaria, which remains a burden of morbidity and mortality in the world, malaria parasite species infect a wide range of hosts including non-human primate, rodent, ungulate, chiroptera, avian, and reptile^1,2^. Ungulate malaria parasites were first reported from antelope and grey duiker in 1913 (*P*. *cephalophi*)^3^, followed by a second parasite reported from grey duikers (*P*. *brucei*), and additional descriptions including goat (*P*. *caprae*), water buffalo (*P*. *bubalis*), mouse deer (*P*. *traguli*), and North American white-tailed deer (*P*. *odocoilei*)^4–9^. Long after these microscopic observations, in 2016, three studies independently reported the first *Plasmodium* DNA sequences from ungulates. Martinsen *et al*.^8^ detected *Plasmodium* parasites (presumptive *P*. *odocoilei*) from white-tailed deer and *Anopheles* mosquitoes in several locations in the United States of America^10^. Boundenga *et al*.^11^ reported *Plasmodium* sequences from duiker antelope in Africa^11^; and Templeton *et al*.^12^ reported *Plasmodium* parasites from water buffalo in southeast Asia, provisionally called *P*. *bubalis* based on an early report in India^4^. In addition, Templeton *et al*.^11^ described a *Plasmodium* sequence isolated from a goat in Zambia^12^. DNA sequences from these samples revealed that the ungulate malaria parasites form a monophyletic clade within the haemosporidian parasites, and branch prior to the clade containing other mammalian and avian/reptile *Plasmodium* parasites^8^. Recently, *Plasmodium* sequences detected from South American pampas deer were shown to be similar to *Plasmodium* spp. in North American white-tailed deer, consistent with a monophyletic grouping of all ungulate malaria parasites^13^.

Goat is a major livestock with a global distribution of 1 billion individuals^14^. Domestic goats are predicted to have originated from the bezoars ibex (*Capra aegagrus aegagrus*) approximately 10,000 years ago, in the Fertile Crescent in southwest Asia including the area of present-day Iran^14^. The first description of a goat malaria parasite, *Laverania caprae*, in Angola in 1923 was from a goat (*Capra aegagrus hircus*) having a submandibular abscess with bacterial infection but no anemia^5^. Images of the parasite were not provided, but morphologies of the ring to schizont stage parasites were described as like *P*. *falciparum*, and gametocytes were termed oval or sickle. The parasite was later renamed *Plasmodium caprae*, but not revisited in the literature until our molecular isolation of a *Plasmodium* sequence in one Zambian goat in 2016. It is not known if the identified *Plasmodium* sequence in the Zambian goat represents a parasite having wider host distribution among African ungulates; versus host specificity to goats, and co-evolution with the host during the process of domestication and global dissemination. Therefore, in this study, we conducted a molecular and morphological surveillance of malaria parasites in goat samples obtained from countries in southeast Asia, west Asia, and Africa, to investigate the distribution of goat malaria parasites, perform comparisons of DNA sequences, and describe intraerythrocytic morphology.

## Results and Discussion

### *Plasmodium caprae* are readily detected in goats in Asian and African countries

**Figure 1:**
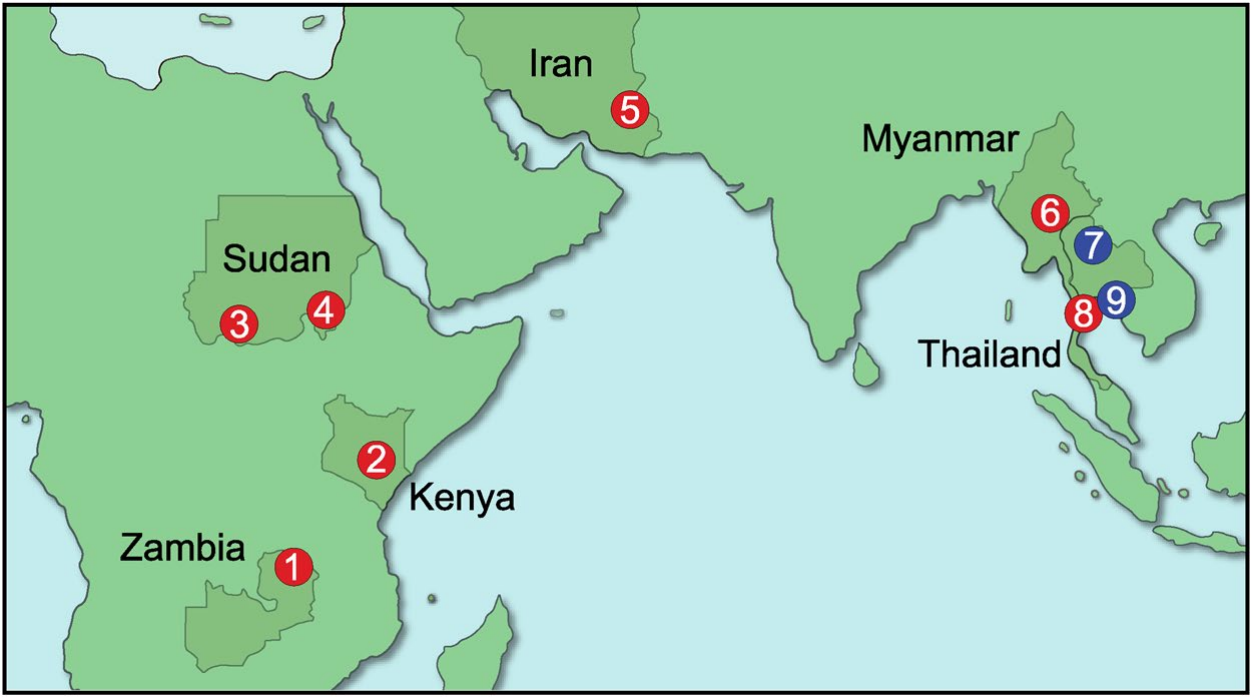
Geographic locations of blood sampling sites. Goat malaria parasites in (1) Zambian samples were collected in 2010, as published^12^. Sampling sites in the present study are in (2) Kiambu and Kitui counties, Kenya (in 2016 and 2017, respectively); (3) West Kordufan state and (4) Blue Nile state, Sudan (2014); (5) Sistan and Baluchestan province, Iran (2016 and 2017); (6) Nay Pyi Taw, Myanmar (2016); and (7) Nan, (8) Phetchaburi, and (9) Chonburi and Rayong provinces, Thailand (2016 and 2017). Sites where *Plasmodium* sequences were detected are shown in red and sites where *Plasmodium* was not detected are shown in blue. The map was made using Adobe Illustrator CC 2017 (Adobe Systems Inc. San José, CA).

A total of 1,299 goat blood samples were collected in 5 countries (Thailand, Myanmar, Iran, Kenya, and Sudan) during 2014 to 2017 and examined by PCR assays targeting the cytochrome b (*cytb*) gene (Fig. 1). *Plasmodium* was detected from all 5 countries (Table 1), and all obtained PCR products (773 bp) matched the sequence of the provisionally called *Plasmodium caprae* reported from a Zambian goat^12^.

**Table 1:** Prevalence of goat malaria parasites.

Country	Location	Period	Number of samples	Positive	Positivity (%)
Thailand	Phetchaburi	May, 2016	126	5	4%
		Jan, 2017	88	0	0%
		July, 2017	100	5	5%
	Chonburi	Jan, 2017	191	0	0%
		July, 2017	100	0	0%
	Rayong	July, 2017	99	0	0%
	Nan	Jan, 2016	190	0	0%
Myanmar	Nay Pyi Taw	Jun, 2016	10	4	40%
Iran	Sistan and Baluchestan	Jan, 2016	24	0	0%
		Jun, 2016	36	0	0%
		Nov, 2016	51	0	0%
		Jul, 2017	89	28	31%
Sudan	West Kurdufan	Apr, 2014	40	1	3%
	Blue Nile	Dec, 2014	76	6	8%
Kenya	Kiambu county	Jun, 2016	13	0	0%
	Kitui county	May, 2017	66	6	9%

In Thailand, the PCR positive cases were found only in Phetchaburi province, and not in Chonburi, Rayong, or Nan provinces. Surveys in Phetchaburi were conducted in May 2016, Jan 2017, and July 2017; and 5 out of 126 (4%), 0 out of 88 (0%) and 5 out of 100 (5%) samples were positive, respectively. The prevalence of *P*. *caprae* in Thai goats was sporadic (0–5%) and much lower than that of *P*. *bubalis* in Thai water buffalo (16–45%)^12^. In Myanmar, a PCR-based survey was conducted in a village in Nay Pyi Taw in June 2016 and 4 out of 10 samples (40%) were positive. The rainy seasons in Thailand and Myanmar are similar, largely dominated by the monsoon, and generally spanning late May or early June to October. *P*. *caprae* positive cases were detected in May and July but not in January, which may reflect a seasonality of *P*. *caprae* infection in these areas, as is known for human malaria infections, although further long-term observations is necessary for the confirmation. This is the first description in Asian countries of goats that are infected with malaria parasites. In Iran, none of the samples collected in January, June, and November 2016 (n = 111) were positive; whereas 28 out of 89 samples (31%) collected in July 2017 were positive, corresponding to the peak season for human malaria in this region^15^. In Kenya, all samples (n = 13) collected in June 2016 were negative, but 6 out of 66 samples (9%) collected in May 2017 were positive. In Sudan, PCR gave positive results for 1 out of 40 samples (3%) collected in West Kordufan state in April 2014, and 6 out of 76 samples (8%) collected in Blue Nile state in December 2014. Together, our data indicate that *P*. *caprae* is widely distributed in Africa (Angola and Zambia from published reports and Sudan and Kenya in this study), west Asia (Iran), and southeast Asia (Myanmar and Thailand)^5,12^. Serological study would be helpful to further clarify the prevalence of the goat malaria infection.

Boundenga *et al*.^11^ detected *Plasmodium* sequences similar to those obtained from African duiker in *Anopheles vinckei*, *A*. *obscurus* and *A*. *gabonensis*^11^. Martinsen *et al*.^10^ reported *Plasmodium* sequences in *A*. *punctipennis* like those obtained from North American white-tailed deer^10^. *Anopheles umbrosus* and *A*. *baezai* (and possibly *A*. *letifer* and *Mansonia crassipes*) were reported as the vectors for mouse deer malaria parasite *P*. *traguli*^1^. These reports suggest that *P*. *caprae* is also transmitted by mosquitoes, although no arthropod vector has been reported for the goat malaria parasite. Given the wide distribution of *P*. *caprae* from Asia to Africa, a variety of local anopheline species are likely able to transmit this pathogen.

Using available data from surveys in which positive cases were detected, the associations of age, gender, or hematocrit levels of goats and *P*. *caprae* positivity were examined. We found no association between goat age and *P*. *caprae* positivity (n = 342; one out of 32 goats <1 years old, 9 out of 138 goats age between 1 and 3 years old, and 31 out of 172 goats ≥ 3 years old were positive). Additional analysis is required to see if infant goats show higher parasite positivity, because we did not collect blood from goats younger than 4 months due to the difficulty to obtain consent from local farmers. There were no associations observed between gender (45 out of 452 female and 3 out of 44 male goats were positive) or hematocrit value (n = 215, odds ratio = 0.92 (95% CI: 0.81–1.04) by logistic regression analysis) and parasite positivity. No statistical difference was detected between pregnancy and parasite positivity using 62 Kenyan female goat samples (1 out of 20 pregnant and 4 out of 42 non-pregnant goats were positive). Larger sample sizes may reveal a clinical significance, if any, of goat malaria infection.

### Microscopic images of goat malaria parasite *Plasmodium caprae*

**Figure 2:**
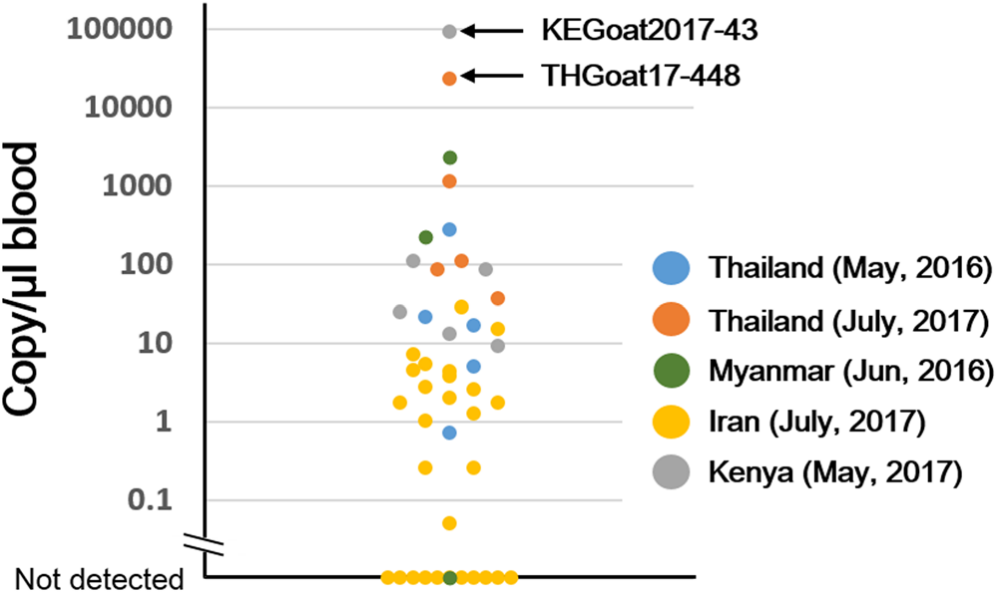
Parasite load in *P*. *caprae* infected goats as measured by qPCR. Copy numbers of mitochondrial DNA per µL of blood are shown. Blue, Thailand in May 2016; orange, Thailand in July 2017; green, Myanmar in June 2016; yellow, Iran in July 2017; and gray, Kenya in May 2017. Values for KEGoat2017-43 and THGoat17-448 samples are indicated.

To evaluate the parasite loads in the infected goats, quantitative PCR (qPCR) targeting mitochondria DNA (mtDNA) was performed. Among 47 samples positive by diagnostic PCR, a Kenyan goat sample collected in 2017 (KEGoat2017–43) showed the highest copy number (~91,000 copy/µL blood equivalent) followed by a Thai sample collected in 2017 (THGoat17-448) with ~23,000 copy/µL blood equivalent (Fig. 2). Since copy numbers of mtDNA in other *Plasmodium* species are estimated to be approximately 30–100 per parasite^16^, the parasitemias of KEGoat2017-43 and THGoat17-448 were estimated to be 0.01–0.03%, and 0.002–0.008%, respectively. The other 45 samples were less than approximately 2,300 copy/µL, indicating that parasitemias in goats by *P*. *caprae* infection are usually very low and barely detectable by microscopy.

**Figure 3:**
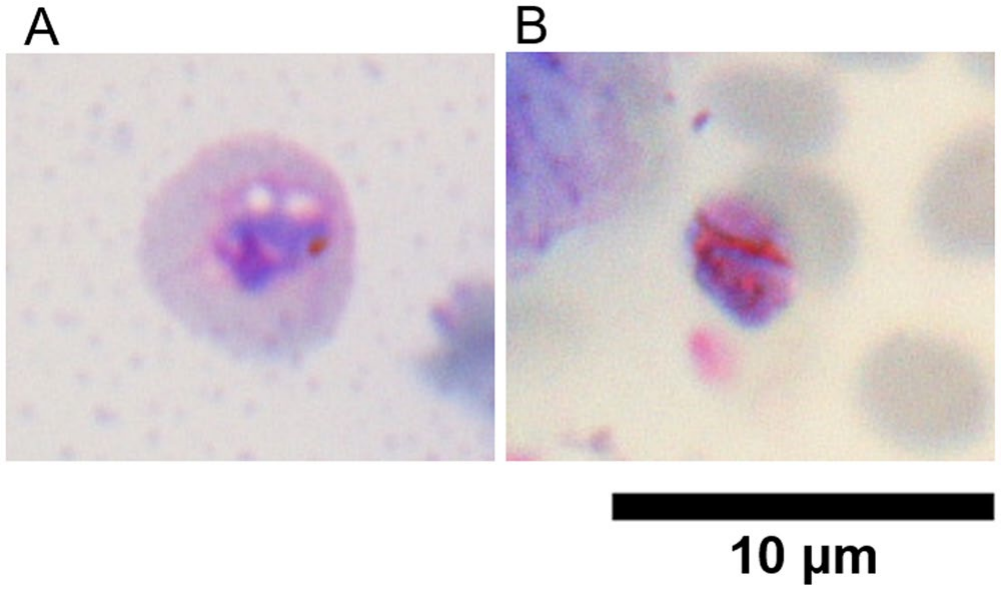
Giemsa-stained images of putative late trophozoites from KEGoat2017-43. (**A**) Putative trophozoite containing one small crystal and two vacuoles. (**B**) Putative trophozoite containing double rod-shaped crystals.

Following a thorough microscopic observation of a blood smear of KEGoat2017–43 and THGoat17-448, we found *Plasmodium*-like organisms in the KEGoat2017-43 sample, with approximately 0.01% parasitemia by eye. Possible amoeboid late trophozoites were characterized harboring a brown pigment, likely hemozoin (Fig. 3). The first image of a putative trophozoite contained one small crystal and two vacuoles, and the size of the erythrocyte infected with this parasite was approximately 6 µm, much larger than un-infected erythrocytes (Fig. 3A). Another image contained double rod-shaped crystals, like the shape of hemozoin of other ungulate malaria parasites such as water buffalo *Plasmodium bubalis*^4^ and grey duiker *Plasmodium cephalophi*^3^. The margin of this erythrocyte infected with a potential trophozoite was not clear, thus it could not be determined if the infected erythrocytes of this pathogen are generally enlarged. Other images of putative parasites are shown in Supplementary Fig. 1. It is formally possible that the found parasites are not *P*. *caprae*, although neither *Babesia* nor *Theileria* DNA were detected from this KEGoat2017-43 sample by a PCR-based diagnostic assay.

### Only one nucleotide substitution was found in the whole mtDNA sequences of *P*. *caprae* among Asian and African isolates

We determined the 5,987 bp whole mitochondrial DNA sequence from the positive samples of each country. One of the sequences from a Kenyan goat (KEGoat2017-102) was identical to the published *Plasmodium* sequence obtained from a Zambian goat (LC090215.1)^12^. The other sequences (3 Thai samples, 2 Myanmar samples, 2 Iranian samples, 2 Sudanese samples [4,909 and 5,115 bp out of 5,987 bp were determined for these samples] and one Kenyan goat sample) had one synonymous substitution at nucleotide (nt) position 4421 (T to A), within the region encoding cytochrome c oxidase subunit I (*coxI*).

To examine the relationship of the *P*. *caprae* sequences with all other known ungulate malaria parasite sequences, including *Plasmodium* from African duiker^11^, North American white-tailed deer^10^, and South American pampas deer^13^, we used 436 bp of partial cytochrome b (*cytb*) sequence. *P*. *caprae* A-type and T-type sequences were monophyletic with a maximum likelihood bootstrap value (BV) of 98 and Bayesian posterior probability (BPP) of 0.91, within a clade containing all ungulate malaria parasites (BV of 100 and BPP of 1.00; Supplementary Fig. 2). When a phylogenetic tree was constructed using whole mitochondrial nucleotide sequences, the *P*. *caprae* A-type sequence was monophyletic with the T-type sequence and formed one clade with the other ungulate malaria parasites, for which whole mitochondrial nucleotide sequences were available, with BV of 100 and BPP of 1.00 (Supplementary Fig. 3), consistent with our previous report^12^. Together, these data confirmed that the A-type and T-type mitochondrial DNA sequences formed one clade within the ungulate malaria parasites. To evaluate the frequency distribution of this single nucleotide polymorphism (SNP) in other areas, we obtained sequences for nt 4,017–4,678 from other positive samples. We found all samples from Thailand, Iran, and Sudan were A-type, whereas both A and T types were found from Kenyan samples (Table 2).

**Table 2:** Genotype at nucleotide position 4421 of mitochondrial DNA.

Country	Nucleotide at 4421	
	A	T
Thailand	9/9 (100%)	0/9 (0%)
Myanmar	3/3 (100%)	0/3 (0%)
Iran	22/22 (100%)	0/22 (0%)
Sudan	6/6 (100%)	0/6 (0%)
Kenya	3/5 (60%)	2/5 (40%)
Zambia*	0/1 (0%)	1/1 (100%)

^*^Zambian data is from a published report^12^.

Goats were proposed to have been domesticated approximately 10,000 years ago in the region called the Fertile Crescent in southwest Asia^14^. From this area, goats were thought to have been introduced to southeast Asia via two routes, one through the Indian subcontinent and the other across the central Asian steppes and China^14,17,18^, although the age of the goat dissemination to Asia has not been clearly elucidated. Introduction to the African continent from southwest Asia is thought to have occurred roughly 7,000 years ago and spread rapidly into the central Sahara and the Ethiopian highlands around 5,000 ~ 6,500 years ago. The domestic goat did not reach southern Africa until approximately 2,000 years ago, likely due to the impact of endemic diseases such as trypanosomiasis^19^. Based on this history of domestic goat dissemination to southeast Asia and Africa from southwest Asia, our favored hypothesis is that the widely detected A-type mtDNA sequence is the original type in the ancestral parasite in the Fertile Crescent and spread broadly, followed by the emergence of the T-type in Africa, which was found only in Kenya and Zambia. However, it is formally possible that the *Plasmodium* parasites were adapted to the goat by a host-switching event after introduction to Africa, then spread to the other regions. Additional SNP information would be useful to provide further insights on this discussion. Nonetheless, the high conservation of mtDNA sequence, with only one nucleotide substitution across approximately 6 kb, suggests that the goat malaria parasite, presumably *Plasmodium caprae*, spread globally with its host domestic goat.

## Conclusion

We performed a PCR surveillance to detect the malaria parasite *P*. *caprae* in goats in Thailand, Myanmar, Iran, Kenya, and Sudan. Putative parasite images were captured from a Giemsa-stained thin blood smear of a Kenyan goat sample. Most positive cases showed very low parasitemia by quantitative PCR and no clear clinical symptoms. The high conservation of the entire mtDNA sequence suggests that the parasite spread globally together with the goat host, rather than multiple events of host switching with local ungulate malaria parasite populations.

## Methods

### Sampling sites and blood collections

Blood samples were collected during the years 2014 to 2017 from indigenous goats in 5 countries; namely, Thailand, Myanmar, Iran, Sudan, and Kenya (see Fig. 1 and Table 1). In Thailand, samples were collected in Phetchaburi Province, which is along the Thai-Myanmar border; Chonburi and Rayong Provinces, in east Thailand; and Nan Province in northern Thailand. Blood was collected aseptically from the jugular vein into BD Vacutainer ACD solution A (Becton, Dickinson and Company). Samples were then divided for making thin blood smears, measuring hematocrit levels, and freezing for later extraction of DNA. Health status and pregnancy information were recorded, if available. There was no preference for blood sampling regarding gender, age, and body weight. The age of goats in Thailand was estimated by tooth numbers and were between 6 months to 8 years old, with 89% (581/655) estimated to be more than 1 year old. Eighty three percent (721/864) were female. In Kenya 13 samples were collected in Ruiru, Kiambu county (approximately 30 km north of Nairobi) and 66 samples in Mwingi, Kitui county (approximately 170 km east of Nairobi). Among these, 62 out of 79 (78%) were females, 22 goats were pregnant, 78 goats were physically healthy, and 1 was sick. In Iran, blood samples were collected in Sistan and Baluchestan province bordering Afghanistan and Pakistan. The ages of goats in Kenya and Iran were obtained from owner farmers. Kenyan and Iranian samples were collected as for the sampling in Thailand. Sudan (West Kurdufan and Blue Nile states) and Myanmar (Nay Pyi Taw) samples were archived DNA samples from the surveillance of other protozoan parasites. Among 116 Sudanese samples, 109 goats (94%) were female. Age information was not obtained for the Sudanese and Myanmar samples. Data are summarized in Table 1.

### DNA extraction

Two hundred µL of blood was treated with 1 mL of 0.15% (w/v) saponin in PBS for 3–5 min and then centrifuged at 10,000 g for 5 min to remove supernatant. The pellets were washed two to three times with PBS until the supernatant turned clear. The pellet was subjected to DNA extraction using a NucleoSpin Blood QuickPure kit (Macherey-Nagel, Germany) or QIAamp DNA Mini kit (Qiagen, Germany) according to the manufacturers’ instructions. DNA was eluted with a final volume of 50 µL (Thai and Iranian samples) or 100 µL (Kenyan, Myanmar, and Sudanese samples). DNA samples were kept at ‒20 °C until use.

### Parasite morphological observations

Two thin blood smears were prepared for each goat sample from Thailand and Kenya. Blood films were fixed with absolute methanol for 3 min and stained with 10% Giemsa solution. Putative *P*. *caprae* images were searched based on the description by de Mello & Paes (1923)^5^ and images of the other ungulate malaria parasites described by Garnham (1966)^1^. Images were captured using a Nikon Digital Sight system with an Eclipse E200 microscope (Nikon, Japan) and a 100× objective lens (oil, N.A. 1.40).

### Parasite DNA amplification and determination of the parasite DNA sequences

DNA sequences containing a part of the *cytb* sequence were amplified by nested PCR with *Plasmodium*-specific universal primers DW2 and DW4 in the primary reaction and with the primers NCYBINF and NCYBINR in the nested PCR reaction, as described^20^. PCR products were subjected to ExoSAP-IT (Thermo Fisher Scientific, Waltham, MA) treatment, and directly sequenced in both direction using BigDye v1.1 and an ABI 3730 DNA Analyzer (Applied Biosystems, Foster City, CA). PCR products from Myanmar goats were cloned into pMD20T-vector (Mighty TA-cloning Kit, TAKARA, Japan), using *E*. *coli* DH5α competent cells. Whole mitochondrial DNA sequences were further determined for three Thai samples (THGoat16-18, THGoat17-435 and THGoat17-448), two Myanmar samples (MMGoat16-2 and MMGoat16-7), two Iranian samples (IRGoat17-15 and IRGoat17-70) and two Kenyan samples (KEGoat17-43 and KEGoat17-102) by direct sequencing of the PCR-amplified DNA fragments as described^12^. Two Sudanese samples (SDGoat16-5 and SDGoat16-24) were also attempted to determine the whole mitochondrial DNA sequences; however, due to sample limitations, only 5.1 kb and 4.9 kb sequences were determined, respectively (nucleotide positions corresponding to the LC326032 1–612, 741–1324, 1402–3102, 3724–3974, 4021–5987 on SDGoat16-5 and 1–1288, 1771–2848, 3006–3973, 4074–4654, 4994–5987 on SDGoat16–24). Before assembly the chromatograms of obtained DNA sequences were visually inspected to ensure that there were no ambiguous nucleotides. Co-infection with *Babesia* or *Theileria* was assayed by PCR using common primers for *Babesia* or *Theileria* and specific primers for *B*. *ovis*^21^ or *T*. *ovis*^22^. Sequence of primers used in this study are shown in Supplementary Table 1.

### Phylogenetic analysis

The obtained whole mtDNA sequences were aligned with known haemosporidian mtDNA sequences using ClustalW software with manual corrections. Mitochondrial DNA sequences used in this study are listed in the Supplementary Table S1 of Templeton *et al*.^12^. Phylogenetic trees were inferred by the maximum likelihood (ML) and Bayesian inference (BI) method. Model analysis using IQ-TREE ver. 1.6.1 indicated that the GTR + I + G model was superior to other models by both Akaike and Bayesian information criterion^23^. Following model analysis, ML analysis was conducted using IQ-TREE 1.6.1 with 1,000 replicates of ultrafast bootstrap analysis. Bayesian posterior probabilities (BPP) were also obtained using MrBayes ver 3.2.6 with eight parallel Metropolis-coupled Markov chain Monte Carlo runs, consisting of one cold and four heated chains with a chain temperature of 0.1, for 3,000,000 generations^24^. Log-likelihood scores and trees with branch lengths were sampled every 1,000 generations and the first 750,000 generations were excluded as burn-in, and the remaining trees were summarized to obtain BPP. The tree was visualized by FigTree ver1.4.3. The phylogenetic tree of 436 bp of *cytb* sequences were also examined with the same method. In addition to the *cytb* sequences obtained from haemosporisdian parasites used in Supplementary Figure 3, nucleotide sequences of *Plasmodium sp*. in the North American white-tailed deer^10^, haemosporidian parasites in the African duiker^11^ and *Plasmodium sp*. in the Brazilian pampas deer^13^ were included in the analysis.

### Quantitative measurement of parasite loads

SYBR Green-based quantitative PCR (qPCR) was performed as described^12^. In brief, qPCR reactions with primers TypeUnivFor and TypeUnivRevii targeting the parasite *cytb* gene were performed on a 7500 Real Time PCR System (Applied Biosystems) with 7500 System SDS software (Applied Biosystems). The pCR-Blunt II-TOPO plasmid containing *P*. *bubalis cytb* sequence was used to make a standard curve to obtain the copy number of the DNA in the tested samples.

### Ethical statement

This study was conducted with the consent of the farm owners and was approved by the Chulalongkorn University Animal Care and Use Committee (No. 1731054). This study was approved by University of Veterinary Science, and the Ministry of Agriculture, Livestock and Irrigation, Myanmar. This study was also conducted with the consent of the farm owners in accordance with the approved guidelines in the other countries described in this study. The project was reviewed and approved by the Institutional Biosafety Committee in accordance with the faculty regulations and policies governing biosafety procedures (IBC approval No. 1631048).

The whole mitochondrial DNA sequence of THGoat16-18 was deposited in DDBJ/ENA/GenBank under the accession number LC326032.

## Electronic supplementary material

Supplementary Information
