# Author Correction: Genetic homogeneity of goat malaria parasites in Asia and Africa suggests their expansion with domestic goat host

**DOI:** 10.1038/s41598-018-25539-w

**Published:** 2018-05-11

**Authors:** Morakot Kaewthamasorn, Mika Takeda, Tawee Saiwichai, Jesse N. Gitaka, Sonthaya Tiawsirisup, Yuhei Imasato, Ehab Mossaad, Ali Sarani, Winai Kaewlamun, Manun Channumsin, Suchart Chaiworakul, Wichit Katepongpun, Surapong Teeveerapunya, Jarus Panthong, Dominic K. Mureithi, Saw Bawm, Lat Lat Htun, Mar Mar Win, Ahmed Ali Ismail, Abdalla Mohamed Ibrahim, Keisuke Suganuma, Hassan Hakimi, Ryo Nakao, Ken Katakura, Masahito Asada, Osamu Kaneko

**Affiliations:** 1 Veterinary Parasitology Research Group, The Veterinary Parasitology Unit, Department of Pathology, Faculty of Veterinary Science, Chulalongkorn University, Bangkok, 10330 Thailand; 2 Animal Vector-Borne Disease Research Group, The Veterinary Parasitology Unit, Department of Veterinary Pathology, Faculty of Veterinary Science, Chulalongkorn University, Bangkok, 10330 Thailand; 3 Department of Protozoology, Institute of Tropical Medicine (NEKKEN), Nagasaki University, 1-12-4 Sakamoto, Nagasaki, 852-8523 Japan; 4 Department of Parasitology and Entomology, Faculty of Public Health, Mahidol University, Bangkok, 10400 Thailand; 5 Department of Clinical Medicine, Mount Kenya University, PO Box, 342-01000, Thika, Kenya; 6 Laboratory of Parasitology, Graduate School of Infectious Diseases, Faculty of Veterinary Medicine, Hokkaido University, Sapporo, 060-0818 Japan; 7 Department of Pathology, Parasitology and Microbiology, College of Veterinary Medicine, Sudan University of Science and Technology, P.O. Box 204, Khartoum, Sudan; 8 Department of Clinical Science, University of Zabol, Veterinary Faculty, PO box +9861335856, Zabol, Iran; 9 School of Agricultural Resources, Chulalongkorn University, Phayathai Rd., Pathumwan, Bangkok 10330 Thailand; 10 Faculty of Veterinary Medicine, Rajamangala University of Technology Tawan-Ok 43 Moo 6 Bangpra, Sriracha District, Chonburi, 20110 Thailand; 11 Faculty of Agriculture and Natural Resources, Rajamangala University of Technology Tawan-Ok 43 Moo 6 Bangpra, Sriracha District, Chonburi, 20110 Thailand; 12 Livestock Office of Phetchaburi Province, Department of Livestock Development, Phetchaburi, 76000 Thailand; 13 Livestock Office of Kaeng Krachan District, Department of Livestock Development, Phetchaburi, 76180 Thailand; 14 Department of Animal Health and Production, School of Pure and Applied Sciences, Mount Kenya University, P O Box, 342-01000, Thika, Kenya; 15 Department of Pharmacology and Parasitology, University of Veterinary Science, Nay Pyi Taw, 15013 Myanmar; 16 Rector Office, University of Veterinary Science, Nay Pyi Taw, 15013 Myanmar; 17 Abrar Research and Training Centre, Abrar University, Mogadishu, Somalia; 18 National Research Center for Protozoan Diseases, Obihiro University of Agriculture and Veterinary Medicine, Obihiro, Hokkaido, 080-8555 Japan; 19 Research Center for Global Agromedicine, Obihiro University of Agriculture and Veterinary Medicine, Obihiro, Hokkaido 080-8555 Japan; 20 Graduate School of Biomedical Sciences, Nagasaki University, 1-12-4 Sakamoto, Nagasaki, 852-8523 Japan

Correction to: *Scientific Reports* 10.1038/s41598-018-24048-0, published online 11 April 2018

In the original version of this Article, there were errors in Affiliation 8 which was incorrectly listed as ‘Department of Clinical Science, Veterinary Faculty, Zabol University, Bonjar Ave, Zabol, 98615-538, Iran.’ The correct affiliation is listed below:

‘Department of Clinical Science, University of Zabol, Veterinary Faculty, PO box +9861335856, Zabol, Iran.’

These errors have now been corrected in the PDF and HTML versions of the Article and in the Supplementary Material.

