# Supplementary material for: Genetic homogeneity of goat malaria parasites in Asia and Africa suggests their expansion with domestic goat host
Source: Sci Rep. 2018 Apr 11;8:5827. doi: 10.1038/s41598-018-24048-0 (PMC5895593; doi:10.1038/s41598-018-24048-0)
Supplement: Supplementary file 1 — Supplementary Information [file 41598_2018_24048_MOESM1_ESM.docx]

**Genetic homogeneity of goat malaria parasites in Asia and Africa suggests their expansion with domestic goat host**

Morakot Kaewthamasorn^1,2^, Mika Takeda^3^, Tawee Saiwichai^1,4^, Jesse N. Gitaka^5^, Sonthaya Tiawsirisup^1,2^, Yuhei Imasato^6^, Ehab Mossaad^7^, Ali Sarani^8^, Winai Kaewlamun^9^, Manun Channumsin^10^, Suchart Chaiworakul^11^, Wichit Katepongpun^11^, Surapong Teeveerapunya^12^, Jarus Panthong^13^, Dominic K. Mureithi^14^, Saw Bawm^15^, Lat Lat Htun^15^, Mar Mar Win^16^, Ahmed Ali Ismail^7^, Abdalla Mohamed Ibrahim^17^, Keisuke Suganuma^18,19^, Hassan Hakimi^3^, Ryo Nakao^6^, Ken Katakura^6^, Masahito Asada^3,20,*^ and Osamu Kaneko^3,20,*^

^1^Veterinary Parasitology Research Group, The Veterinary Parasitology Unit, Department of Pathology, Faculty of Veterinary Science, Chulalongkorn University, Bangkok, 10330 Thailand

^2^Animal Vector-Borne Disease Research Group, The Veterinary Parasitology Unit, Department of Veterinary Pathology, Faculty of Veterinary Science, Chulalongkorn University, Bangkok, 10330 Thailand

^3^Department of Protozoology, Institute of Tropical Medicine (NEKKEN), Nagasaki University, 1-12-4 Sakamoto, Nagasaki, 852-8523, Japan.

^4^Department of Parasitology and Entomology, Faculty of Public Health, Mahidol University, Bangkok 10400 Thailand

^5^Department of Clinical Medicine, Mount Kenya University, PO Box 342-01000, Thika, Kenya.

^6^Laboratory of Parasitology, Graduate School of Infectious Diseases, Faculty of Veterinary Medicine, Hokkaido University, Sapporo 060-0818, Japan

^7^Department of Pathology, Parasitology and Microbiology, College of Veterinary Medicine, Sudan University of Science and Technology, P.O. Box 204, Khartoum, Sudan.

^8^Department of Clinical Science, University of Zabol, Veterinary Faculty, PO box +9861335856, Zabol, Iran

^9^School of Agricultural Resources, Chulalongkorn University, Phayathai Rd., Pathumwan, Bangkok, 10330, Thailand

^10^Faculty of Veterinary Medicine, Rajamangala University of Technology Tawan-Ok 43 Moo 6 Bangpra, Sriracha District, Chonburi 20110, Thailand

^11^Faculty of Agriculture and Natural Resources, Rajamangala University of Technology Tawan-Ok 43 Moo 6 Bangpra, Sriracha District, Chonburi 20110, Thailand

^12^Livestock Office of Phetchaburi Province, Department of Livestock Development, Phetchaburi 76000, Thailand

^13^Livestock Office of Kaeng Krachan District, Department of Livestock Development, Phetchaburi 76180, Thailand

^14^Department of Animal Health and Production, School of Pure and Applied Sciences, Mount Kenya University, P O Box 342-01000 Thika, Kenya.

^15^Department of Pharmacology and Parasitology, University of Veterinary Science, Nay Pyi Taw 15013, Myanmar

^16^Rector Office, University of Veterinary Science, Nay Pyi Taw 15013, Myanmar

^17^Abrar Research and Training Centre, Abrar University, Mogadishu, Somalia.

^18^National Research Center for Protozoan Diseases, Obihiro University of Agriculture and Veterinary Medicine, Obihiro, Hokkaido 080-8555, Japan.

^19^Research Center for Global Agromedicine, Obihiro University of Agriculture and Veterinary Medicine, Obihiro, Hokkaido 080-8555, Japan.

^20^Graduate School of Biomedical Sciences, Nagasaki University, 1-12-4 Sakamoto, Nagasaki, 852-8523, Japan.

Correspondence and requests for materials should be addressed to M.A. (email: masada@nagasaki-u.ac.jp) or O.K. (email: okaneko@nagasaki-u.ac.jp).

**Additional Information**

**Supplementary Table 1. Primers used in this study**

| Primer name | sequence | reference |
| --- | --- | --- |
| DW2 | TAATGCCTAGACGTATTCCTGATTATCCAG | [1] |
| DW4 | TGTTTGCTTGGGAGCTGTAATCATAATGTG | [1] |
| NCYBINF | TAAGAGAATTATGGAGTGGATGGTG | [1] |
| NCYBINR | CTTGTGGTAATTGACATCCAATCC | [1] |
| TypeUnivFor | CGTGCTAAAGGTTTAACAC | [2] |
| TypeUnivRevii | TATAATACTGGATCACCAGC | [2] |
| Bbo-F | TGGGCAGGACCTTGGTTCTTCT | [3] for *B. ovis* |
| Bbo-R | CCGCGTAGCGCCGGCTAAATA | [3] for *B. ovis* |
| TSsr 170F | TCGAGACCTTCGGGT | [4] for *T. ovis* |
| TSsr 670R | TCCGGACATTGTAAAACAAA | [4] for *T. ovis* |
| F14 | CTCATTCATATCCAAGCCTC | in this study |
| RevNCYF | CACCATCCACTCCATAATTCTCTTA | in this study |
| F16 | CCAACATTTGCTGGTGATCCAG | in this study |
| R12 | GGAATACGTCTAGGCATTAC | in this study |
| Babesia_Sense | GTTCCAAGGAATTTGAATTCTAATTGG | in this study |
| Babesia_As_2 | GCAAACTTCCCGGCTAAACTT | in this study |
| Babesia_Sense_2 | GCTCAATGTGTTTCTTTTTG | in this study |
| Theil_Sense_2 | AAAGGTATGTGGTATTCTAGTAA | in this study |
| Theil_As_2 | CTTTTGTTGGAAACACTTTTAATGTTGC | in this study |
| Theil_Sense_3 | CTTCATAGAGATGGAAGTTCAAATCCATT | in this study |

**
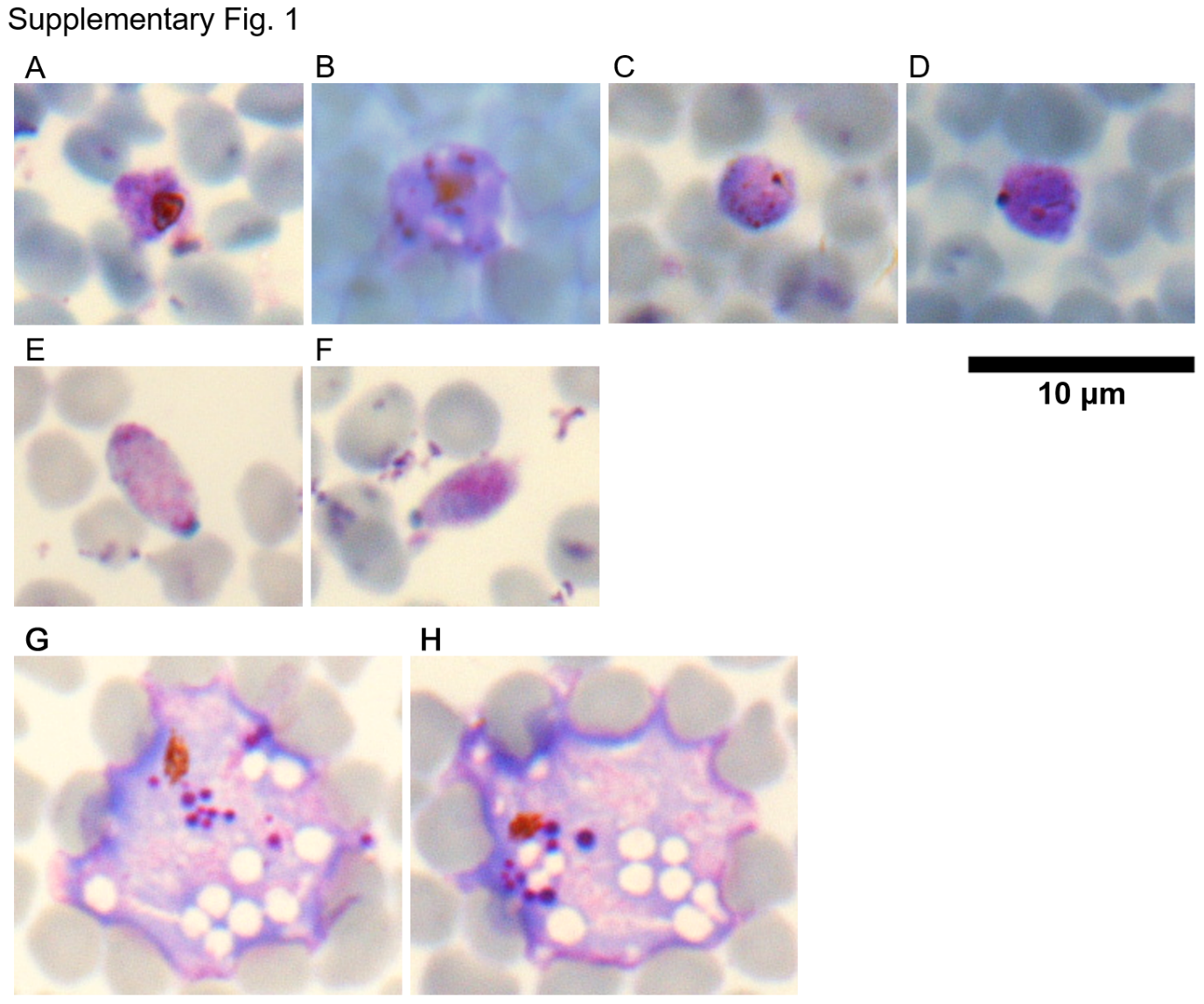
**

**Supplementary Figure 1. Images obtained from Giemsa-stained thin blood smear of KEGoat2017-43**.

(A) Putative trophozoite containing a large pigment. (B) Putative not fully segmented schizont. Multiple nuclei and one large brown pigment with many smaller pigments were visible. (C, D) Putative early gametocytes of approximately 3.5 µm in diameter were seen, having a round shape and cytosol with a blueish stain. Nuclei in pink color were seen in a wide area within the cell and having no clear margin. Many brown pigments of unequal size were scattered in the cytosol with 1 prominent brown dot of approximately 0.5 µm in size located on the periphery. (E, F) Putative more matured gametocytes. These putative gametocytes showed an oval shape of 3 µm wide and 5.5 - 6 µm long. Cytosol were stained light pink with multiple tiny brown pigments. One prominent brown dot was seen at the one end of the long axis, and a similar character might be seen in putative early gametocytes. de Mello and Paes (1923)^5^ described that the macrogametocyte was oval or sickle-shape (5 - 6 x 2 - 2.5 μm) with pigments dispersed throughout the cell; and that the microgametocyte was smaller, oval (3.5 - 4.5 x 1 - 2 μm), and the pigment was, in general, focused on two poles. The oval shape and the size of putative gametocytes in E and F matches the description of the macrogametocytes by de Mello and Paes (1923)^5^, with a discrepancy in the location and number of the pigments. (G, H) Macrophage-like cells with a clear brown pigment, resembling a malaria pigment, with several purple dots and vacuoles in the cytosol.


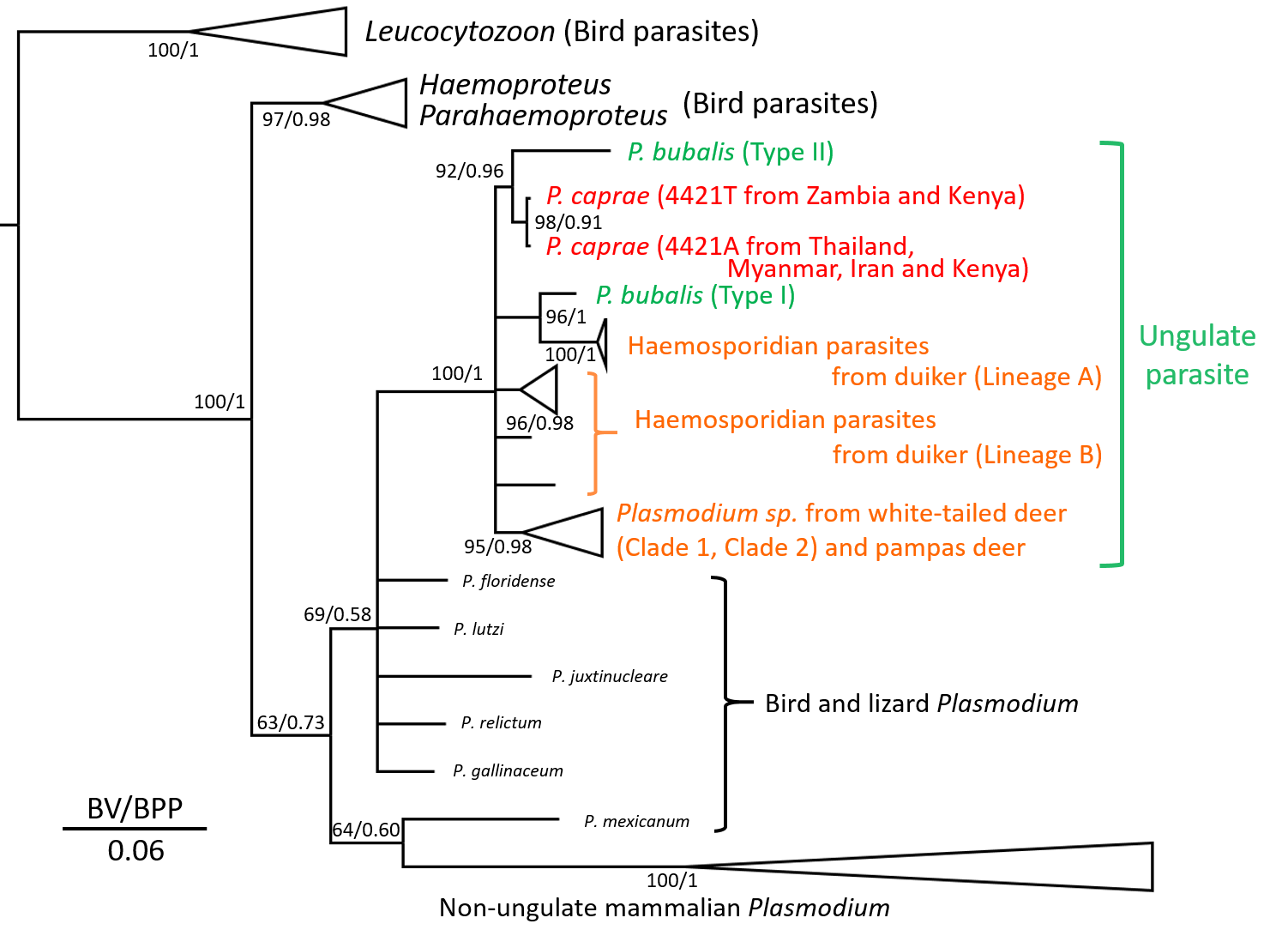


**Supplementary Figure 2. Monophyletic relationship of all known ungulate *Plasmodium spp.* including *Plasmodium caprae* using 436 bp *cytb* nucleotide sequence**

The tree was constructed using partial nucleotide sequences of *cytb* (436 bp) by the maximum likelihood and Bayesian method based on the GTR + I + G model. Bootstrap values (BV) for maximum likelihood method with 1,000 replicates of ultrafast bootstrap analysis and Bayesian posterior probability (BPP) are indicated for each internal branch. The *cytb* sequences of *Plasmodium bubalis*, Zambian *P. caprae* and non-ungulate haemosporidian parasites used in this study are listed in Supplementary Table S1 of Templeton et al. (2016)^2^. Nucleotide sequences of *Plasmodium sp.* in the North American white-tailed deer were based on Table S4 of Martinsen et al. (2016)^6^. Nucleotide sequences of *Plasmodium sp.* in the South American pampas deer were based on Asada et al. (2018)^7^. Nucleotide sequences of haemosporidian parasites in the African duiker were based on Supporting Information Table A of Boundenga et al. (2016)^8^. The *cytb* sequences obtained from *Plasmodium sp.* in the North American white-tailed deer (KU133755, KU133748, KU133754, KU133751, KU133749, KU133750, KU133752 and KU133753) and the nucleotide sequences of haemosporidian parasites in the African duiker (KT367840, KT367833, KT367822, KT367835, KT367828, KT367830, KT367841, KT367819, KT367839, KT367837, KT367832, KT367834, KT367817, KT367821, KT367842, KT367838, KT367823, KT367826, KT367820, KT367829, KT367825, KT367836, KT367831, KT367824 and KT367827) were used for this analysis. The length for the substitutions/site (0.06) is indicated.


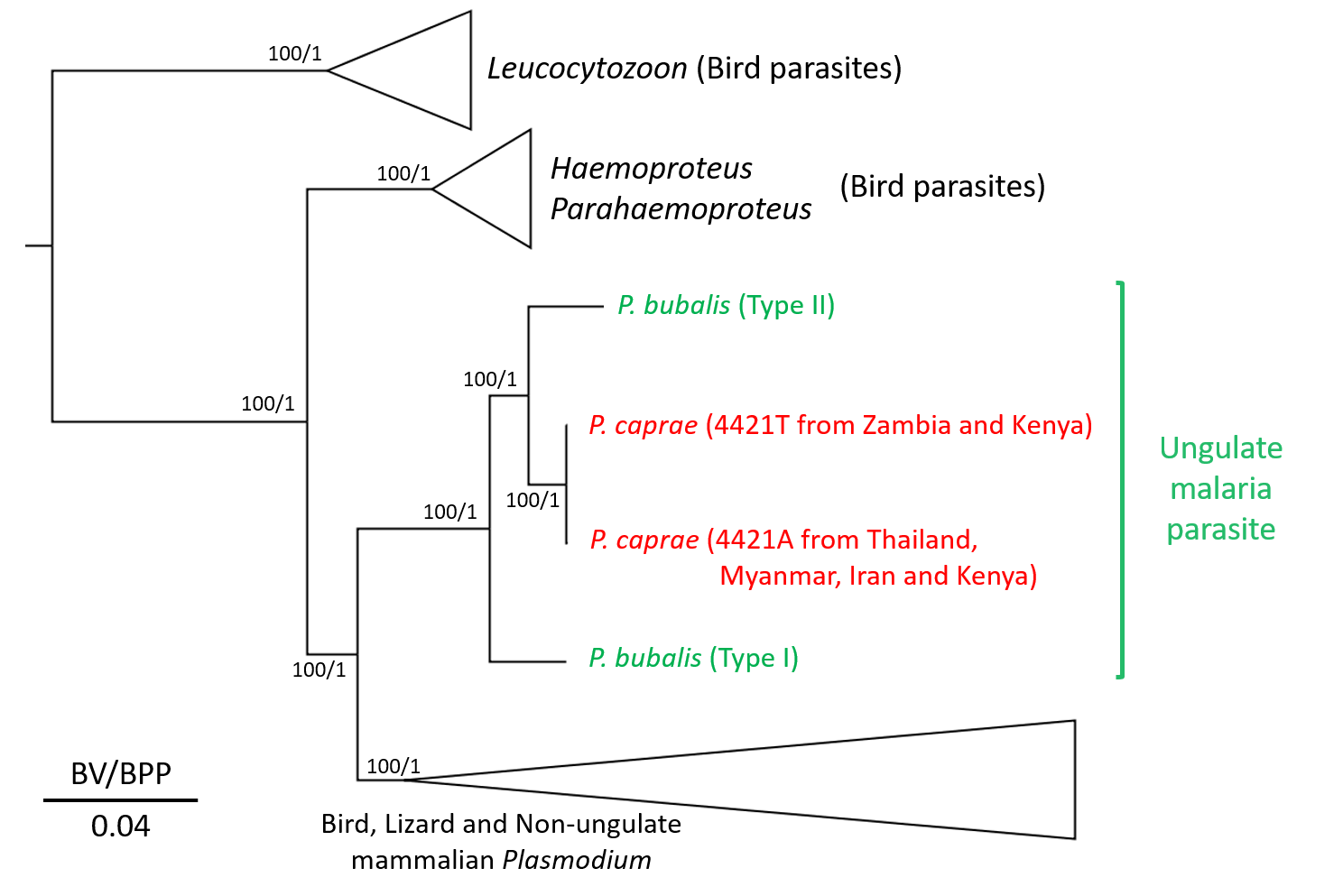


**Supplementary Figure 3. Phylogenetic location of *Plasmodium caprae* sequences in Haemosporidia using whole mitochondrial nucleotide sequence**

The tree was constructed using whole mitochondrial nucleotide sequences by the maximum likelihood and Bayesian method based on the GTR + I + G model. Bootstrap values (BV) for maximum likelihood method with 1,000 replicates of ultrafast bootstrap analysis and Bayesian posterior probability (BPP) are indicated for each internal branch. The compositions of collapsed clades are *Leucocytozoon* (*L. fringillinarium, L. majoris, and L. sabrasezi*); *Haemoproteus* and *Parahaemoproteus* (*Haemoproteus* sp. jb1.JA27, *Haemoproteus* sp. jb2.SEW5141, and *Parahaemoproteus vireonis*); and bird, lizard and non-ungulate mammalian *Plasmodium* (*P. gallinaceum, P. relictum, P. juxtinucleare, P. lutzi, P. floridense, P. mexicanum, P. falciparum, P. vivax, P. malariae, P. ovale, P. coatneyi, P. cynomolgi, P. fieldi, P. gonderi, P. inui, P. knowlesi, P. fragile, P. simiovale, P. simium, P. hylobati, P. reichenowi, P. billicollinsi, P. billbrayi, P. berghei, P. chabaudi, P. vinckei,* and *P. yoelii*). Mitochondrial DNA sequences used in this study are listed in Supplementary Table S1 of Templeton et al. (2016)^2^. Nucleotide positions containing indels were excluded from the alignment of whole mitochondrial nucleotide sequences (5889 ~ 6684 bp) and 5509 bp were used for analysis. The length for the substitutions/site (0.04) is indicated.

**References**

1. Keymer, I. F. Studies on *Plasmodium* (*Vinckeia*) *cephalophi* of the grey duiker (*Sylvicapra grimmia*). *Ann. Trop. Med. Parasitol*. **60**, 129-138 (1966).

2. Templeton, T. J. et al. Ungulate malaria parasites. *Sci. Rep.* **6**, 23230 (2016).

3. Aktaş, M., Altay, K., Dumanli, N. Development of a polymerase chain reaction method for diagnosis of *Babesia ovis* infection in sheep and goats. *Vet. Parasitol.* **133**, 277-281 (2006).

4. Aktas, M., Altay, K., Dumanli, N. PCR-based detection of *Theileria ovis* in *Rhipicephalus bursa* adult ticks. *Vet. Parasitol.* **140**, 259-263 (2006).

5. de Mello, F. & Paes. S. Sur une plasmodiae du sang des chèvres. C.r. *séanc. Soc. Biol.* **88**, 829–830 (1923).

6. Martinsen, E. S. et al. Hidden in plain sight: Cryptic and endemic malaria parasites in North American white-tailed deer (*Odocoileus virginianus*). *Sci. Adv.* **2**, e1501486 (2016).

7. Amills, M., Capote, J. & Tosser-Klopp, G. Goat domestication and breeding: a jigsaw of historical, biological and molecular data with missing pieces. *Anim. Genet.* **48**, 631-644 (2017).

8. Boundenga, L. et al. Haemosporidian parasites of antelopes and other vertebrates from Gabon, Central Africa. *PLoS ONE* **11**, e0148958 (2016).
